# Orthodontic tooth movement enhancing bony apposition in alveolar bony defect: a case report

**DOI:** 10.1186/1757-1626-2-116

**Published:** 2009-02-03

**Authors:** Kyoko Hibino, Ricky WK Wong

**Affiliations:** 1 Discipline of Orthodontics, Faculty of Dentistry, the University of Hong Kong, 2/F Prince Philip Dental Hospital, 34 Hospital Road, Hong Kong SAR, PR China

## Abstract

**Introduction:**

Prevalence of complications from orthognathic surgery is relatively low but if it happens it is vital to manage the post complication bony defect appropriately.

**Case Presentation:**

This case report describes a 20-year-old gentleman who suffered from a complication from a bimaxillary orthognathic surgery. A bone grafting was carried out to repair the bony defect from the surgery but it was unsuccessful. A non-invasive technique employing the use of very light orthodontic force with a laceback stainless steel ligature is described and a successful space closure with an improvement in the periodontal condition and bone apposition has been shown.

**Conclusion:**

This technique can be considered if orthodontic tooth movement is needed across a deficient alveolar ridge.

## Introduction

Bone defect in the alveolus region may occur after orthognathic surgery if the apposition of bone segment is not ideal or if there is postoperative infection causing loss of sequestrum. Such defects render postsurgical orthodontic tooth movement impossible across the defects and result in incomplete space closure and poor occlusion.

This case report describes the non-invasive technique employing the use of very light orthodontic force in tooth movement in a region of alveolar bone defect.

## Case presentation

A 20-year-old gentleman had a bimaxillary orthognathic surgery in 2005. He was diagnosed with bimaxillary dentoalveolar protrusion. In the maxilla, Wunderer and Posterior LeFort I osteotomies with extractions of upper first premolars were carried out. In the mandible, lower segmental (Hofer) osteotomy with extractions of lower first premolars, and advancement and reduction genioplasty were carried out.

At two weeks after surgery, there was wound dehiscence between the upper left canine and second premolar region. One month post surgery, a cleft like defect distal to the upper left canine was noted.

Upon initial orthodontic assessment 6 months after surgery, a suppuration site was detected distal to the upper left canine with a probing depth of 8 mm. Removal of sequestrum and granulation tissue was carried out and the suppuration stopped and gingival recession 5 mm was noted with reduced probing depth to 3 mm. However an alveolar ridge deficiency remained between the upper left canine and second premolar region with no active inflammation.

The patient was referred to the oral surgeon to have the alveolar bone graft prior to the orthodontic tooth movement. Ramus bone graft was carried out in 2007 to the bony defect. Unfortunately, bone regeneration distal to the upper left canine did not occur.

**Figure 1:**
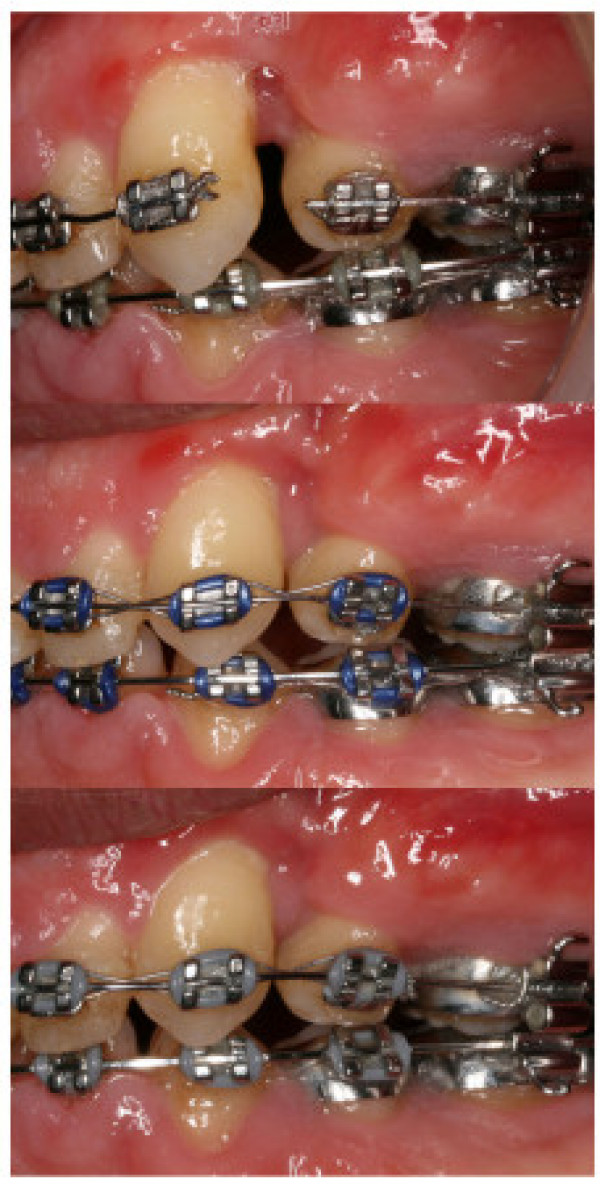
**Intra-oral photograph showing the alveolar cleft**. **Top**: Intra-oral photograph showing the alveolar cleft between upper left canine and second premolar with gingival recession, bony defect and spacing. **Middle**: Intra-oral photograph 6 months after the initial treatment showing the laceback with a 0.010 inch soft stainless steel ligature. **Bottom**: Intra-oral photograph 1 year later showing the laceback with a 0.010 inch soft stainless steel ligature and the closure of the space with the improvement of the gingival recession.

**Figure 2:**
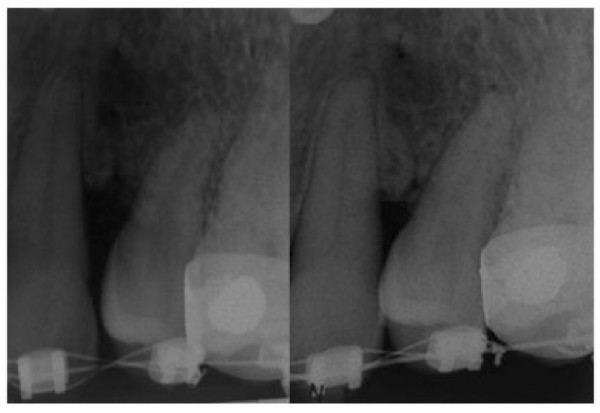
**Radiographs showing the alveolar cleft**. **Left**: Periapical radiograph showing the alveolar cleft between upper left canine and second premolar with triangular bony defect. **Right**: Periapical radiograph 1 year later showing the apposition of bone in the alveolar cleft between upper left canine and second premolar region.

In order to close the space between the upper left canine and second premolar without worsening the periodontal attachment and bony defect, a very light orthodontic force was applied more than three months after the bone grafting. 014 inch copper NiTi wire with stainless steel ligature laceback from upper right second molar to upper left second premolar (0.010 inch stainless steel wire) was used with the canine bracket rebonded to more incisal position to attempt to intrude it at the same time as distalising canine root and mesialising upper left second premolars because of angulations of the bracket slots (Fig. 1). The laceback was tightened every visit which was approximately one month apart. Following one year of gentle orthodontic force application with the laceback ligature, an improvement in the bony defect was noted clinically and radiographically (Fig. 1 and Fig. 2). The gingival recession has reduced to about 3 mm from 5 mm, the probing was 5 mm and bone has been deposited between the two teeth.

## Discussion

The usual protocol for the management of closure of alveolar cleft is to use bone grafting [1]. In addition, it has been shown that a high success rate in the treatment of residual alveolar cleft when tooth movement was followed by bone grafting [2].

However, in this case, the bone grafting was not successful and a different approach has been adapted and was demonstrated to work successfully in this patient.

Bone filling was observed radiographically following the application of a gentle orthodontic force with a laceback ligature. It is postulated that the angulation of the bracket slot caused a gentle deformation of the copper NiTi archwire, with in turn transmitted a very light force to move the roots across the bone defect. This in turn induced bone remodeling and bone formation around the root and the bone defect. It also induced angiogenesis to bring in pluripotential mesenchymal cells to the bone defect site for tissue formation.

Conflicting results regarding the effect of orthodontic tooth movement on periodontal healing has been described in the past. Enhance periodontal and bone regeneration by orthodontic tooth movement towards a bony defect was reported [3]. However, no such effect was also reported [4].

Verdimon *et al*. [5] revealed that the total bony apposition was 6.5 fold larger with the orthodontic tooth movement into the surgical bony defects in rats. They concluded that orthodontic tooth movement is a stimulating factor of bone apposition. Furthermore, it was also shown that enhanced bone healing following orthodontic movement where the defect involved periodontal structures [6]. This case report is also in agreement with the above findings that it is possible for apposition of bone into bony defect by orthodontic movement.

In this case report, a laceback was used to apply the gentle force. Laceback technique in preadjusted appliance was first described by McLaughlin and Bennett [7]. It is constructed of either 0.009 or 0.010 inch soft stainless steel tied in a figure of 8 from the most distally incorporated molar to the canine bracket. They were utilized to control crown position during leveling and aligning of the teeth with the archwire with the advantage of canine distalisation without tipping.

It was shown that the laceback ligatures are effective for canine distalisation [8]. In a randomized controlled clinical trial investigating the effectiveness of the laceback ligatures showed no statistical or clinical difference in the anteroposterior or vertical position of the lower labial segment or in the relief of labial segment crowding and the use of laceback ligatures creates a statistically and clinically significant increase in the loss of posterior anchorage, through mesial movement of the lower first molars [9]. In our case report, the laceback was used not in the way that McLaughlin and Bennett were intended but to apply gentle force to close the bony defect. The ability to retract canine gradually and the loss of posterior anchorage is favorable in this situation to close the space.

It was important to apply light orthodontic force to the periodontally compromised teeth with bony defect. In periodontally compromised teeth, the loss of alveolar bone results in the centre of resistance of the involved teeth moving apically, thus light controlled forces is important to minimize further attachment loss, tipping movement and root resorption [10]. In addition, the upper left canine was intruded slightly because it is shown that gingival recession improved following intrusion of the tooth [11].

## Conclusion

This case report has demonstrated the effective space closure and movement of teeth into the bony defect with bony apposition to the site with the light orthodontic force from the use of a laceback ligature. The technique can be considered when tooth movement across a bone deficient alveolar ridge is needed.

## Competing interests

The authors declare that they have no competing interests.

## Authors' contributions

KH has made substantial contribution to the clinical treatment of the patient, acquisition of the data and has been involved in drafting the manuscript. RW has made substantial contributions to application of laceback technique in tooth movement in bone defects, revising the manuscript critically for important intellectual content and have given final approval of the version to be published. Both authors read and approved the final manuscript.

## Authors' information

**Kyoko Hibino **BDS (U.K.); Postgraduate student (MOrth)

Ricky W.K. Wong* BDS (HK), MOrth (HK), PhD (HK), FRACDS, MOrthRCS (Edin), FHKAM (Dental Surgery), FCDSHK (Orthodontics); Associate professor in Orthodontics

KH is a postgraduate student in Master of Orthodontics, Faculty of Dentistry, the University of Hong Kong.

RWKW is an associate professor in Orthodontics, Faculty of Dentistry, the University of Hong Kong.

## Consent

Written informed consent was obtained from the patient for publication of this case report and accompanying images. A copy of the written consent is available for review by the Editor-in-Chief of this journal.

## References

[B1] Bajaj A Wongworawat A Punjabi A Management of alveolar clefts J Craniofac Surg 2003 14 840 846 10.1097/00001665-200311000-00005 14600625

[B2] Bergland O Semb G Abyholm F Elimination of the redifual alveolar cleft by secondary bone grafting and subsequent orthodontic treatment Cleft Palate Craniofac 1986 23 175 205 3524905

[B3] Nevins M Wise R Use of orthodontic therapy to alter infrabony pockets. 2 Int J Periodontics Restorative Dent 1990 10 198 207 2098349

[B4] Wennström J BL BS Nyman S Thilander B Periodontal tissue response to orthodontic movement of teeth with infrabony pockets Am J Orthod Dentofacial Orthop 1993 103 313 319 10.1016/0889-5406(93)70011-C 8480696

[B5] Vardimon A Nemcovsky C Dre E Orthodontic Tooth Movement Enhances Bone Healing of Surgical Bony Defects in Rats J Periodontol 2001 72 858 864 10.1902/jop.2001.72.7.858 11495132

[B6] Nemcovsky C Beny L Shanberger S Feldman-Herman S Vardimon A Bone apposition in surgical bony defects following orthodontic movement: a comparative histomorphometric study between root- and periodontal ligament-damaged and periodontally intact rat molars J Periodontol 2004 75 1013 1019 10.1902/jop.2004.75.7.1013 15341361

[B7] McLaughlin R Bennett J The transition from standard edgewise to preadjusted appliance systems J Clin Orthod 1989 23 142 153 2606968

[B8] Sueri M Turk T Effectiveness of laceback ligatures on maxillary canine retraction Angle Orthod 2006 76 1010 1014 10.2319/100605-351 17090165

[B9] Irvine R Power S The effectiveness of laceback ligatures: a randomized controlled clinical trial J Orthod 2004 31 303 11 10.1179/146531204225020606 15608345

[B10] Johal A Ide M Orthodontics in the adult patient, with special reference to the periodontally compromised patient Dent Update 1999 26 101 114 10528549 10.12968/denu.1999.26.3.101

[B11] Re S Cardaropoli D Abundo R Corrente G Reduction of gingival recession following orthodontic intrusion in periodontally compromised patients Orthod Craniofac Res 2004 7 35 39 10.1111/j.1601-6343.2004.00277.x 14989753

